# Genetic determinants of telomere length and risk of common cancers: a Mendelian randomization study

**DOI:** 10.1093/hmg/ddv252

**Published:** 2015-07-02

**Authors:** Chenan Zhang, Jennifer A. Doherty, Stephen Burgess, Rayjean J. Hung, Sara Lindström, Peter Kraft, Jian Gong, Christopher I. Amos, Thomas A. Sellers, Alvaro N.A. Monteiro, Georgia Chenevix-Trench, Heike Bickeböller, Angela Risch, Paul Brennan, James D. Mckay, Richard S. Houlston, Maria Teresa Landi, Maria N. Timofeeva, Yufei Wang, Joachim Heinrich, Zsofia Kote-Jarai, Rosalind A. Eeles, Ken Muir, Fredrik Wiklund, Henrik Grönberg, Sonja I. Berndt, Stephen J. Chanock, Fredrick Schumacher, Christopher A. Haiman, Brian E. Henderson, Ali Amin Al Olama, Irene L. Andrulis, John L. Hopper, Jenny Chang-Claude, Esther M. John, Kathleen E. Malone, Marilie D. Gammon, Giske Ursin, Alice S. Whittemore, David J. Hunter, Stephen B. Gruber, Julia A. Knight, Lifang Hou, Loic Le Marchand, Polly A. Newcomb, Thomas J. Hudson, Andrew T. Chan, Li Li, Michael O. Woods, Habibul Ahsan, Brandon L. Pierce

**Affiliations:** 1 Department of Public Health Sciences,; 2 Center for Cancer Epidemiology and Prevention,; 3 Department of Medicine,; 4 Department of Human Genetics, The University of Chicago, Chicago, IL, USA,; 5 Section of Biostatistics and Epidemiology,; 6 Center for Genomic Medicine, Department of Community and Family Medicine, Geisel School of Medicine, Dartmouth College, Lebanon, NH, USA,; 7 Department of Public Health and Primary Care,; 8 Centre for Cancer Genetic Epidemiology, Department of Public Health and Primary Care, University of Cambridge, Cambridge, UK,; 9 Lunenfeld-Tanenbaum Research Institute of Mount Sinai Hospital, Toronto, Canada,; 10 Department of Epidemiology, Harvard School of Public Health, Boston, MA, USA,; 11 Division of Public Health Sciences, Fred Hutchinson Cancer Research Center, Seattle, WA, USA,; 12 Department of Cancer Epidemiology, H. Lee Moffitt Cancer Center, Tampa, FL, USA,; 13 Department of Genetics, QIMR Berghofer Medical Research Institute, Brisbane, Australia,; 14 Department of Genetic Epidemiology, University Medical Center, Georg-August-University Göttingen, Göttingen, Germany,; 15 Division of Epigenomics and Cancer Risk Factors, DKFZ, German Cancer Research Center,; 16 Translational Lung Research Center Heidelberg (TLRC-H), Member of the German Center for Lung Research (DZL), Heidelberg, Germany,; 17 International Agency for Research on Cancer, Lyon, France,; 18 Division of Genetics and Epidemiology, Institute of Cancer Research, Sutton, Surrey, UK,; 19 Division of Cancer Epidemiology and Genetics, National Cancer Institute, National Institutes of Health, U.S. Public Health Service, Bethesda, MD, USA,; 20 Institute of Epidemiology I, Helmholtz Zentrum München, German Research Center for Environmental Health, Neuherberg, Germany,; 21 The Institute of Cancer Research, Sutton, UK,; 22 Royal Marsden National Health Service (NHS) Foundation Trust, London and Sutton, UK,; 23 Warwick Medical School, University of Warwick, Coventry, UK,; 24 Institute of Population Health, University of Manchester, Manchester, UK,; 25 Department of Medical Epidemiology and Biostatistics, Karolinska Institute, Stockholm, Sweden,; 26 Department of Preventive Medicine, Keck School of Medicine, University of Southern California/Norris Comprehensive Cancer Center, Los Angeles, CA, USA,; 27 Molecular Genetics/Laboratory Medicine and Pathobiology, Mount Sinai Hospital, University of Toronto, Toronto, Canada,; 28 Centre for Epidemiology and Biostatistics, Melbourne School of Population and Global Health, The University of Melbourne, Parkville, Australia,; 29 Division of Cancer Epidemiology, German Cancer Research Center (DKFZ), Heidelberg, Germany,; 30 Cancer Prevention Institute of California, Fremont, CA, USA,; 31 Department of Epidemiology, University of North Carolina School of Public Health, Chapel Hill, NC, USA,; 32 Kreftregisteret, Cancer Registry of Norway, Oslo, Norway,; 33 Stanford University School of Medicine, Stanford, CA, USA,; 34 USC Norris Comprehensive Cancer Center, University of Southern California, Los Angeles, CA, USA,; 35 Ontario Cancer Genetics Network, Fred A. Litwin Center for Cancer Genetics, Samuel Lunenfeld Research Institute, Mount Sinai Hospital, Toronto, ON, Canada,; 36 Division of Epidemiology, Dalla Lana School of Public Health, University of Toronto, Toronto, ON, Canada,; 37 Samuel Lunenfeld Research Institute, Mount Sinai Hospital, Toronto, ON, Canada,; 38 Department of Preventive Medicine, Northwestern University, Chicago, IL, USA,; 39 Epidemiology Program, University of Hawaii Cancer Center, Honolulu, HI, USA,; 40 Department of Epidemiology, University of Washington School of Public Health, Seattle, WA, USA,; 41 Ontario Institute for Cancer Research, Toronto, ON, Canada,; 42 Division of Gastroenterology, Massachusetts General Hospital and Harvard Medical School, Boston, MA, USA,; 43 Channing Division of Network Medicine, Brigham and Women's Hospital and Harvard Medical School, Boston, MA, USA,; 44 Department of Family Medicine and Community Health, Case Western Reserve University, Cleveland, OH, USA and; 45 Discipline of Genetics, Faculty of Medicine, Memorial University, Newfoundland and Labrador, Canada

## Abstract

Epidemiological studies have reported inconsistent associations between telomere length (TL) and risk for various cancers. These inconsistencies are likely attributable, in part, to biases that arise due to post-diagnostic and post-treatment TL measurement. To avoid such biases, we used a Mendelian randomization approach and estimated associations between nine TL-associated SNPs and risk for five common cancer types (breast, lung, colorectal, ovarian and prostate cancer, including subtypes) using data on 51 725 cases and 62 035 controls. We then used an inverse-variance weighted average of the SNP-specific associations to estimate the association between a genetic score representing long TL and cancer risk. The long TL genetic score was significantly associated with increased risk of lung adenocarcinoma (*P* = 6.3 × 10^−15^), even after exclusion of a SNP residing in a known lung cancer susceptibility region (*TERT-CLPTM1L*) *P* = 6.6 × 10^−6^). Under Mendelian randomization assumptions, the association estimate [odds ratio (OR) = 2.78] is interpreted as the OR for lung adenocarcinoma corresponding to a 1000 bp increase in TL. The weighted TL SNP score was not associated with other cancer types or subtypes. Our finding that genetic determinants of long TL increase lung adenocarcinoma risk avoids issues with reverse causality and residual confounding that arise in observational studies of TL and disease risk. Under Mendelian randomization assumptions, our finding suggests that longer TL increases lung adenocarcinoma risk. However, caution regarding this causal interpretation is warranted in light of the potential issue of pleiotropy, and a more general interpretation is that SNPs influencing telomere biology are also implicated in lung adenocarcinoma risk.

## Introduction

Telomeres are DNA–protein complexes at chromosome ends that help maintain genome stability by protecting DNA from damage and fusion. The DNA component is a six-base TTAGGG repeat sequence that shortens with each cell division. In differentiated cells, telomere shortening eventually leads to loss of telomere protection and genome instability, typically triggering cell senescence or programmed cell death (1). In stem and progenitor cells, the telomerase enzyme elongates telomeres, enabling prolonged cell survival (2). Telomerase is also activated in >90% of human tumors (3), which typically have short telomeres (a potential cause of genome instability), thus promoting proliferation and survival (4).

The critical role of telomeres and telomerase in carcinogenesis has led to the hypothesis that short telomere length (TL) is a risk factor for cancer (5). Indeed, short relative TL measured in surrogate tissues, such as peripheral blood cells, has been associated with increased risk for lung (6,7), ovarian (8), colorectal (9) and breast cancers (10,11) in epidemiological studies (with the interpretation that blood TL predicts cancer risk because it is a proxy for TL in cancer-prone tissues). However, such associations are not consistent across all cancers or even within cancer types, with some studies reporting null, U-shaped or positive associations (11–16). Furthermore, due to the retrospective nature of case–control studies from which many of these association estimates are obtained, telomere shortening that occurs after diagnosis, potentially due to treatment (17,18) or disease progression, can result in biased estimates of the association between TL and cancer risk (6,8,14).

Genome-wide association (GWA) studies have identified several genomic regions containing variants associated with TL in peripheral blood cells (19–21), including the *TERT* (telomerase reverse transcriptase) region (5p15.33). Furthermore, GWA studies of cancer risk have observed that variants in the *TERT* region influence risk for multiple cancer types, including breast (22), colorectal (23), lung (24), prostate (24) and ovarian (22) cancer, although these associations do not appear to all be driven by the same causal variant. In light of this evidence indicating an important role for telomeres in carcinogenesis, we undertook a comprehensive examination of associations between genetic determinants of TL and cancer risk.

In this work, we describe the associations between nine TL-associated genetic variants and risk for five cancer types (breast, lung, colorectal, ovarian and prostate), using data from the Genetic Associations and Mechanisms in Oncology (GAME-ON) network of consortia for post-GWA research. In addition, we estimate the association between a multi-variant TL score and cancer risk, which corresponds to the effect of TL on cancer risk under Mendelian randomization assumptions (25). However, this interpretation requires caution because the validity of the Mendelian randomization assumptions (such as the absence of pleoitropy) cannot be proven.

Because genotype–phenotype associations are not vulnerable to biases caused by reverse causation or confounding by environment, the Mendelian randomization approach used in this study is an attractive approach for estimating relationships between TL and cancer risk.

## Results

The Genetic Associations and Mechanisms in Oncology (GAME-ON) Consortium is a network of five consortia focused on cancers of the breast, colon, lung, ovary and prostate. The GAME-ON network represents 33 GWA studies contributing data on >51 000 cancer cases and >62 000 controls (26). Samples sizes for each cancer type and subtype are listed in Table 1.

**Table 1.** Sample sizes for cancer types included in the Genetic Associations and Mechanisms in Oncology (GAME-ON) consortium. Details on the GAME-ON Network and the contributing GWA studies have been previously described (26) (http://epi.grants.cancer.gov/gameon/)

Cancer type	Cases	Controls	GWA studies^a^
Breast			
All	15 748	18 084	11
ER-negative	4939	13 128	8
Colorectal	5100	4831	6
Lung^b^			
All	12 160	16 838	9
Adenocarcinoma	3718	15 871	9
Squamous	3422	16 015	9
Ovarian			
All	4369	9123	3
Clear-cell	356	9123	3
Endometrioid	715	9123	3
Serous	2556	9123	3
Prostate			
All	14 160	12 724	6
Aggressive	4450	12 724	6

^a^Not including studies from the Genetics and Epidemiology of Colorectal Cancer Consortium (GECCO).

^b^Subtypes listed do not represent all subtypes within cancer type.

### Association estimates for individual SNPs

**Figure 1.**
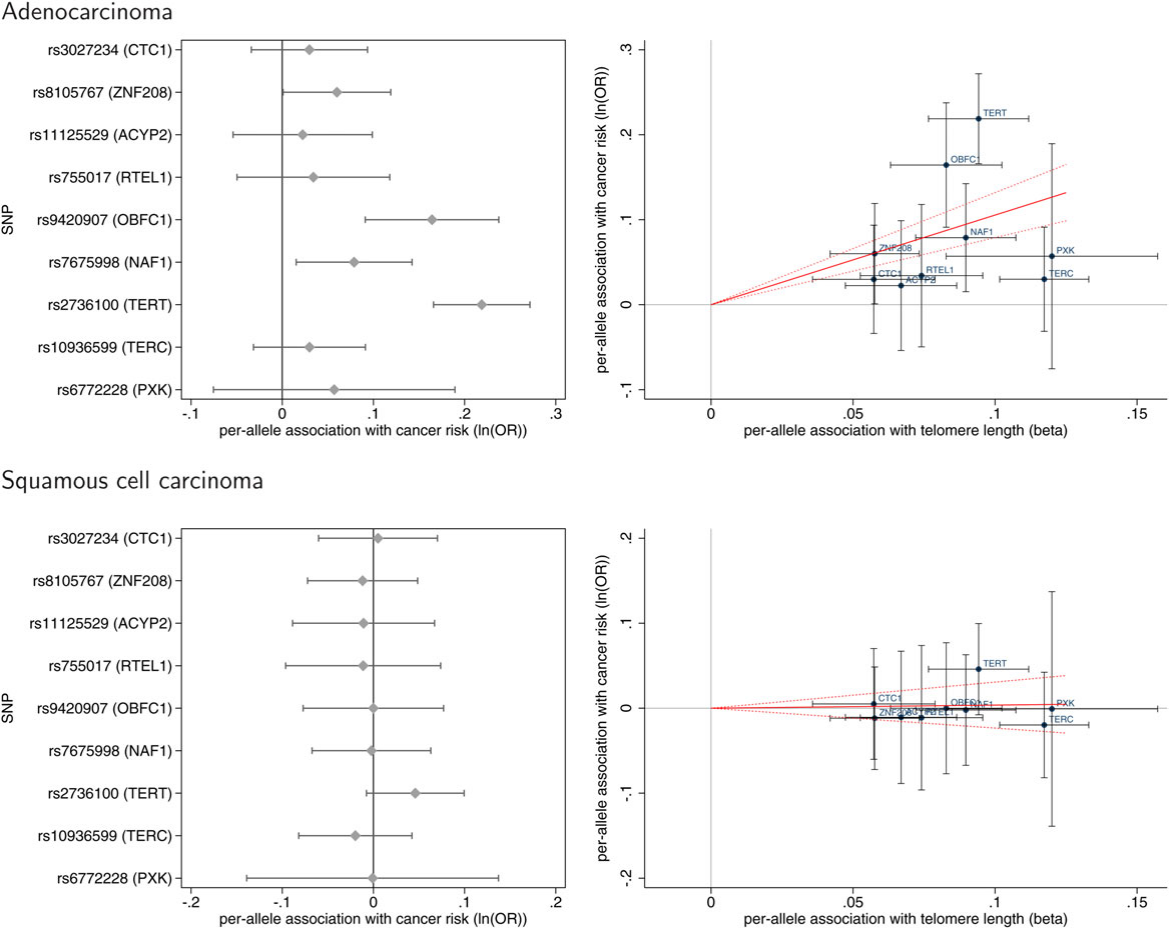
Forest plots (left) and scatter plots (right) of associations between TL-associated SNPs and risk for lung adenocarcinoma (top) and squamous cell carcinoma (bottom). Forest plots show association estimates (with horizontal bars indicating 95% CI) for the ‘long telomere’ allele of each SNP with cancer risk. SNPs are ordered by increasing magnitude of association with TL. Scatter plots show the per-allele association with cancer risk plotted against the per-allele association with kb of TL (with vertical and horizontal black lines showing 95% cCI for each SNP). The scatter plot is overlaid with the Mendelian randomization estimate (slope of red solid line with dotted lines showing 95% CI) of the effect of TL on cancer risk

Based on the existing literature, we identified nine SNPs showing genome-wide significant associations (*P* < 5 × 10^−8^) with TL in GWA studies (19–21). From these prior papers we obtained the identifier for the lead SNP at each reported locus as well as the ‘long TL’ allele, association estimate for the ‘long’ allele (in terms of kb of TL per allele), and the standard error and *P*-value for each SNP's association with TL (Table 2). We estimated associations between each of the nine TL-associated SNPs and risk for each of the five common cancer types and subtypes in the GAME-ON study, shown as forest plots in Supplementary Material, Figure S1. Of note, for all nine SNPs, the long TL allele had an OR > 1 for lung adenocarcinoma, with four of the nine associations being statistically significant (*P* < 0.05) (Fig. 1, top left). In contrast, no TL-associated SNP was significantly associated with squamous cell carcinoma of the lung (Fig. 1, bottom left). Prostate cancer risk also showed nominally significant positive associations with the long TL alleles for three of the nine SNPs (*P* < 0.05) (Supplementary Material, Fig. S1).

**Table 2.** Characteristics of genetic variants associated with TL as reported in prior GWA studies

SNP identifier	Chromosome	Locus	‘Long’ allele	*β* estimate^a^	*P*-value	Source
rs10936599	3	*TERC*	C	0.117	2.5 × 10^−31^	Codd *et al*. (19)
rs2736100	5	*TERT*	C	0.094	4.4 × 10^−19^	Codd *et al*. (19)
rs7726159^b^	5	*TERT*	A	0.073	4.7 × 10^−17^	Pooley *et al*. (21)
rs7675998	4	*NAF1*	G	0.090	4.3 × 10^−16^	Codd *et al*. (19)
rs9420907	10	*OBFC1*	C	0.083	6.9 × 10^−11^	Codd *et al*. (19)
rs6772228	3	*PXK*	T	0.120	3.9 × 10^−10^	Pooley *et al*. (21)
rs8105767	19	*ZNF208*	G	0.058	1.1 × 10^−9^	Codd *et al*. (19)
rs755017	20	*RTEL1*	G	0.074	6.7 × 10^−9^	Codd *et al*. (19)
rs412658^c^	19	*ZNF676*	T	0.050	9.8 × 10^−9^	Mangino *et al*. (20)
rs3027234	17	*CTC1*	C	0.057	2.3 × 10^−8^	Mangino *et al*. (20)
rs11125529	2	*ACYP2*	A	0.067	4.5 × 10^−8^	Codd *et al*. (19)

^a^Reported in kb telomere per ‘long’ allele.

^b^In linkage disequilibrium (*r*^2^ = 0.382) with rs2736100 of the *TERT* locus, excluded from all analyses.

^c^In linkage disequilibrium(*r*^2^ = 0.704) with rs8105767 of the *ZNF208* locus, excluded from all analyses.

### Mendelian randomization estimates based on multi-SNP scores

We estimated the associations between a multi-SNP TL score and risk for each cancer (Table 3) using a previously described Mendelian randomization approach that obtains an estimate using an inverse-variance weighted average of SNP-specific associations (27,28) (see Materials and Methods). Of note, we identified a highly statistically significant association between long TL and increased risk of lung adenocarcinoma with an odds ratio (OR) of 2.78 per 1 kb increase TL [95% confidence interval (CI) 2.16, 3.58; *P* = 6.3 × 10^−15^]. However, we observed no such association for squamous cell carcinoma of the lung. Associations for these two lung cancer subtypes are displayed in Figure 1 (right) as solid red lines (slope = ln(OR)) overlaid on the association estimates for the nine SNPs that were used to generate the OR. A positive slope indicates that longer TL is associated with increased cancer risk, while a negative slope indicates that longer TL is associated with decreased risk. The correlation (*r*) between the magnitude of the SNPs' associations with TL and the magnitude of the SNPs' associations with adenocarcinoma risk was 0.17. Other than lung adenocarcinoma, no other cancer types showed a statistically significant association with the multi-SNP score. However, prostate cancer risk showed suggestive evidence of positive association with long TL with a Mendelian randomization OR of 1.21 per 1 kb increase in TL (95% CI 0.99, 1.46; *P* = 0.06). Scatter plots for all cancer types are displayed in Supplementary Material, Figure S2.

**Table 3.** ORs of cancer risk per 1000 bp increase in TL according to a multi-SNP TL score using the inverse-variance weighted method (left) and the likelihood method (right)

	Inverse-variance weighted method			Likelihood method		
Cancer type	OR	95% CI	*P*-value	OR	95% CI	*P*-value
Breast						
All	1.02	0.86, 1.21	0.82	1.02	0.86, 1.21	0.81
ER-negative	1.05	0.81, 1.38	0.70	1.05	0.80, 1.38	0.70
Colorectal	1.25	0.92, 1.69	0.15	1.26	0.92, 1.71	0.15
Lung						
All	1.65	1.39, 1.96	1.3 × 10^−8^	1.67	1.40, 2.00	1.3 × 10^−8^
Adenocarcinoma	2.87	2.20, 3.74	6.3 × 10^−15^	3.03	2.29, 4.01	8.2 × 10^−15^
Squamous	1.04	0.79, 1.36	0.79	1.04	0.79, 1.36	0.79
Ovarian						
All	1.13	0.87, 1.47	0.37	1.13	0.87, 1.48	0.36
Clear-cell	1.65	0.78, 3.51	0.19	1.68	0.78, 3.61	0.19
Endometrioid	1.30	0.75, 2.24	0.35	1.30	0.75, 2.25	0.35
Serous	1.19	0.86, 1.65	0.30	1.19	0.86, 1.66	0.29
Prostate						
All	1.21	0.99, 1.46	0.06	1.22	1.00, 1.48	0.06
Aggressive	1.10	0.83, 1.45	0.52	1.10	0.83, 1.46	0.51

Additional age- and sex-stratified analyses were conducted for overall lung cancer, with findings indicating similar estimates for younger subjects (≤50 years old) (OR = 1.95; 95% CI 1.19, 3.21; *P* = 0.008) and older subjects (>50 years old) (OR = 1.78; 95% CI 1.47, 2.15; *P* = 2.98 × 10^−9^) and similar estimates for men (OR = 1.72; 95% CI 1.38, 2.14; *P* = 1.06 × 10^−6^) and women (OR = 1.99; 95% CI 1.42, 2.77; *P* = 5.3 × 10^−5^).

In addition to the inverse-variance weighted approach for obtaining Mendelian randomization estimates, we also used a likelihood-based Mendelian randomization method (28). Both methods produced very similar estimates for all cancer types, although the lung adenocarcinoma estimates varied more between the two methods compared with the other cancer types (Table 3).

### Sensitivity analyses

The estimates reported above can only be interpreted as the causal effect of average TL on cancer risk when the Mendelian randomization assumptions are valid—namely, when (1) the SNPs from the literature are truly predictive of TL in the cancer-prone tissue, (2) the SNPs are not associated with other factors (confounders) that influence both TL and cancer risk and (3) the SNPs only affect cancer risk through their effects on TL, i.e. there are no alternative causal pathways by which the SNPs influence cancer risk. Violation of any of the assumptions can result in a biased causal estimate for the effect of TL on cancer risk. We performed sensitivity analyses in which SNPs were excluded from the multi-SNP score based on potential violation of these assumptions.

To assess a potential violation of the first assumption, an additional analysis was performed after excluding the SNP near the *PXK* region (rs6772228), which may be a false-positive association evidenced by its lack of plausible biological explanation, and the lack of consistency in its association with TL across several study sites (21). This ‘strict’ analysis resulted in a notable difference only for prostate cancer risk, which now showed a statistically significant estimate (OR 1.27; 95% CI 1.03, 1.54; *P* = 0.02) (Supplementary Material, Table S1).

To assess potential violation of the second and third assumptions, an additional analysis was performed including only SNPs for which the magnitude of association with cancer risk appeared to be proportional to the magnitude of association with TL. Under Mendelian Randomization assumptions two and three, the ratio of the SNP-cancer association to the SNP–TL association for each SNP should be similar for all SNPs used in the SNP score. An inflation of the ratio for any single SNP may be an indication that the SNP exerts a pleiotropic effect on cancer that is unrelated to its effect on TL. Deviation from this expectation is tested using a goodness-of-fit test (29,30), in which SNPs that exhibit evidence of pleiotropy due to an inflated SNP–cancer to SNP–TL ratio can be detected and excluded from Mendelian randomization analyses (see Materials and Methods).

The exclusion of SNPs that failed the goodness-of-fit test (Supplementary Material, Table S2) resulted in two notable findings (Supplementary Material, Table S1). First, exclusion of the SNPs in the *TERT* and *CTC1* regions from the SNP set used for overall prostate cancer resulted in a stronger, statistically significant association (OR = 1.45; 95% CI 1.18, 1.82; *P* = 7.9 × 10^−4^). Second, the exclusion of the SNP in the *TERT* region from the SNP set used for lung adenocarcinoma resulted in a somewhat attenuated association, but the association remained statistically significant: (OR = 2.00, 95% CI 1.48, 2.70, *P* = 6.6 × 10^−6^). Lung adenocarcinoma had a significant heterogeneity test statistic (Supplementary Material, Table S2), which likely explains why there was a difference in the estimates obtained using the inverse-variance weighted method and the likelihood method noted earlier; the optimization algorithm of the likelihood-based method can have poor convergence when the heterogeneity statistic is strongly significant (28). This difference in estimates between methods is eliminated after excluding the *TERT* SNP (rs2736100) that drives the heterogeneity in association estimates for lung adenocarcinoma.

## Discussion

In this analysis of cancer risk across five cancer-prone organs, we observed that a multi-SNP score for long telomeres was significantly associated with increased risk of lung adenocarcinoma (but not squamous cell carcinoma) and suggestively associated with increased risk of prostate cancer. We did not observe an association between the multi-SNP score and risk of breast, colorectal or ovarian cancer (including subtypes). Under Mendelian randomization assumptions, the associations reported here can be interpreted as effects of TL on cancer risk, although caution regarding such an interpretation is warranted because the validity of these assumptions (such as the absence of pleiotropy) cannot be proven. Our results were consistent using two different analytic approaches. After performing sensitivity analyses in which SNPs were excluded from the multi-SNP score based on potential violation of Mendelian randomization assumptions, the association with prostate cancer risk became statistically significant. In addition, the exclusion of the SNP in the *TERT* region (a known susceptibility locus for lung cancer) from the lung adenocarcinoma analysis resulted in an attenuated but still highly statistically significant association, indicating that the observed association is not solely driven by the SNP in the *TERT* region. Even after dropping the three SNPs showing nominally significant (*P* < 0.05) association with lung adenocarcinoma (in *TERT*, *OBFC1* and *NAF1*) the association is still significant (OR = 1.54, *P* = 0.018).

Several epidemiologic studies have examined the association between leukocyte TL and lung cancer risk. Three retrospective case–control studies reported an association between long TL and decreased lung cancer risk in US and Korean subjects (7,31,32). However, a fourth stratified retrospective case–control study showed a positive association between TL and adenocarcinoma risk but an inverse association for squamous cell carcinoma (33). In two studies with prospective TL measurement, long TL was found to be associated with increased overall lung cancer risk among Caucasian male smokers (34) and East Asian female never-smokers (13), while a large Danish general population study found no association (12). In a pooled analysis of three prospective cohort studies including the two aforementioned studies and a third study conducted in the USA, long telomeres were associated with increased lung cancer risk, and the association was present in adenocarcinoma while absent in squamous cell carcinoma (35). Consistent with findings from the prospective studies and the stratified case–control study, we observed a positive association between the long TL SNP score and lung cancer risk corresponding to an OR of 1.65 per 1000 bp TL for overall lung cancer (*P* = 1.3 × 10^−8^) and an OR of 2.87 per 1000 bp TL for lung adenocarcinoma (*P* = 6.3 × 10^−15^) (assuming valid Mendelian randomization assumptions). Seow *et al*. (35) reported the risk of overall lung cancer between the lowest quartile and highest quartile as OR = 1.86 (95% CI 1.33–2.62), and the risk of adenocarcinoma cancer as OR = 2.52 (95% CI 1.38–4.60). While our estimates are not directly comparable to these prior estimates due to differences in the scale of the TL variable (kb vs. quartiles), we used a simple simulation to show that our estimates are similar to these prior estimates. We simulated normally distributed TL variables with a mean of 6000 bp and standard deviations ranging from 400 to 700 bp based on values observed from the prior literature (36–38). The difference between the mean values for quartiles one and four ranged between 1018 and 1781 bp. We then rescaled our ORs to correspond to a difference in 1018–1781 bp rather than 1000 bp. The resulting estimates are OR = 1.68–2.94 for overall lung cancer risk between the lowest and highest quartiles, and OR = 2.92–5.11 for lung adenocarcinoma risk between the lowest and highest quartiles. These are comparable with the estimates reported by Seow *et al*. The observed heterogeneity in our association estimates is likely due to the two subtypes being biologically distinct, having previously been characterized as having different genetic susceptibilities (39), unique gene expression profiles (40), distinct molecular features (41) and different patterns of chromosomal imbalance (42).

The Asian female non-smokers among whom a TL-lung cancer association was observed (13) were also recently studied to evaluate seven TL-associated SNPs in relation to lung cancer risk (43). Consistent with our findings, the risk score for long TL was associated with an increase in lung cancer. Furthermore, their stratified analyses suggested a stronger association among younger individuals ( <60 years old) and significant associations for both adenocarcinoma and squamous cell subtypes. In contrast, our stratified analyses produced similar estimates by age and different estimates by subtype. Potential factors driving these differences in findings may be differences in ancestry or differences in the etiology of subtypes between the two study populations.

A protective effect of short TL on lung cancer risk has a biologically plausible explanation, as short telomeres could protect against cancer by triggering cell senescence or programmed cell death in the presence of functional cell cycle checkpoints and intact apoptotic pathways (44). Conversely, long telomeres may enable additional rounds of cell division, allowing more opportunities for the accumulation of somatic mutations that promote carcinogenesis, resulting in greater susceptibility to malignant transformation (45,46). The association between long TL SNPs and increased risk has also been previously observed for melanoma (47), with a proposed mechanism being that long telomeres increase the proliferative duration of cells, thus delaying senescence and allowing further mutations to occur (48).

Cigarette smoking is a potential confounder in many epidemiologic studies of TL and cancer due to its correlation with short TL (49). However, our study utilizes genetic variants associated with TL as proxy for actual measured TL, and is therefore not subject to the potential confounding effects of smoking and other exposures. TL-outcome confounders such as smoking would only introduce bias if the genetic variants used in the score are also associated with smoking behavior, as it would be a violation of the second Mendelian randomization assumption. Smoking-related violation of Mendelian randomization assumptions (e.g., SNPs that influence TL through smoking behavior) is unlikely due to the lack of an association between the TL SNP score and squamous cell carcinoma of the lung, which would be expected to have a stronger association with smoking-related SNPs than adenocarcinoma (50), and due to the lack of evidence that these SNPs influence smoking behavior based on prior GWA studies.

There are few published studies on TL and prostate cancer risk. In a small retrospective case–control study (51) and two prospective studies (12,52), no statistically significant association was observed. In contrast, we observe a suggestive association between long TL and increased risk for prostate cancer, an association that increases in significance in the context of the ‘goodness-of-fit’-based sensitivity analyses. This finding warrants further investigation.

Results from prior studies of TL and colorectal cancer risk are also inconsistent. An inverse association between TL and colorectal cancer risk was described in two case–control studies (9,14), while a U-shaped association (16) and three null-associations (12,14,15) were observed in prospective studies. Consistent with the previous null-findings, our results show no significant association between TL and colorectal cancer risk, despite the inclusion of the *TERC* rs10936599 SNP, which was previously reported to be associated with both increased TL and increased risk of colorectal cancer (53). The null findings were also consistent in an analysis of data from the Genetics and Epidemiology of Colorectal Cancer Consortium (GECCO) (54) (Supplementary Material, Fig. S3).

Although one prospective study showed evidence for association between long TL and increased breast cancer risk (12), two different meta-analyses of TL and breast cancer risk based on multiple retrospective and prospective studies concluded there was no overall evidence of association (6,8). Our findings for overall breast cancer are in agreement with these prior null studies. In addition, we observe no association between the TL SNP score and ER-negative breast cancer risk. For ovarian cancer, two prior case–control studies observed an association between longer telomeres and decreased risk (55,56), one case–control study reported no association (57), while a prospective study also reported no association (12). Our results for overall ovarian cancer, as well as three subtypes, provide no evidence of association with TL-associated SNPs. This lack of association is observed despite the inclusion of SNP rs2736100 located in the *TERT* region, which showed a nominally significant association with the serous subtype of ovarian cancer (*P* = 0.023) and is in high LD (*r*^2^ = 0.8) with a SNP previously observed to be associated with ovarian cancer (22)*.* Estrogen has been demonstrated in experimental studies to have positive effects on telomerase activity (58), and in epidemiologic studies estrogen has been shown to have a positive association with TL (59). With estrogen as a potential confounder of the association between TL and ER-positive breast and ovarian cancers, it will likely be difficult to parse out the specific role of TL in estrogen-related cancer risk in epidemiological studies. However, Mendelian randomization estimates such as those reported here will not be biased due to confounding by estrogen level. These multi-SNP null findings are similar to what Pooley et al. (21) also observed while investigating individual TL SNP associations with breast, ovarian and prostate cancer risks.

Although it is not possible to prove the validity of the Mendelian randomization assumptions, it is possible to conduct sensitivity analyses to protect against some potential violations of these assumptions. To address a potential violation of the first assumption—that the SNPs are associated with TL in our study population—we conducted analyses excluding *PXK* SNP rs6772228, whose association with TL has been questioned due to the lack of consistency in its association across several study sites (21). After exclusion, the results were essentially unchanged. For overall prostate cancer, however, the association became statistically significant, lending support to the hypothesis that long TL is associated with an increased risk of prostate cancer.

To address potential violations of the assumption that the SNPs do not have effects on cancer risk independent of their effects on TL, we re-estimated the associations between the TL SNP score and cancer after stepwise removal of potentially problematic SNPs from the SNP set using a goodness-of-fit test of the proportionality of the SNPs' associations with TL and cancer risk. These exclusions resulted in some attenuation of the association with lung adenocarcinoma, but did not substantially alter our conclusions. For prostate cancer, the association with the multi-SNP score became statistically significant after excluding *TERT* SNP rs2736100 and *CTC1 SNP* rs3027234. The heterogeneity in association of SNPs in *TERT* and *CTC1* identified by the goodness-of-fit test suggests potential pleiotropic effects of these genetic variants through mechanisms other than TL. The association between *TERT* SNPs and breast and ovarian cancer risks via pathways other than TL has been previously observed (22), and potential extra-telomeric roles have previously been suggested for both telomerase (60) and *CTC1* (61), providing a plausible biological basis for excluding the *TERT* and *CTC1* SNPs from our analysis. It is important to note however, that these secondary sensitivity analyses are data-driven, and are presented as a supplement to the primary analyses that include all nine SNPs.

There are several limitations of this work. The summary-level data did not allow for analyses stratified by covariates of interest such as sex and age (with the exception of lung cancer, for which TRICL conducted stratified analyses). Additionally, our analysis assumed a log-linear association between TL and cancer risk, and the existence of a non-linear (e.g. U-shaped) association may limit our ability to detect an association. Our estimates generated using Mendelian randomization are unbiased only if the SNPs analyzed do not affect cancer risk through causal pathways other than those involving TL. This assumption cannot be proven; however, our confidence in the validity of this assumption is strengthened by the fact that our primary finding is robust to the exclusion of SNPs with potential pleiotropic actions based on prior evidence (*TERT*) and a goodness-of-fit test (Supplementary Material, Tables S1, S2) (although it is possible that the goodness-of-fit test is underpowered to identify pleiotropic effects). Our power to detect associations is limited by the small variance in measured TL explained by SNPs used in this analysis (1–2%) (62), although the GAME-ON Network provides very large sample sizes that enable the detection of strong-to-moderate associations (Supplementary Material, Table S3). Finally, we cannot confirm that genetic determinants of leukocyte TL also predict tissue-specific TL due to the lack of tissue-based TL measures in GWA studies. A potential consequence of selecting SNPs lacking tissue-specific association with TL would be a bias toward the null, reducing our power to detect associations using TL SNP scores. However, correlations between TL measured in blood and TL measured in lung (63,64) and other tissues (65) have been reported (*r* = 0.35–0.84), consistent with the assumption that SNPs predict TL across multiple tissues. Systematic studies on other tissue types are needed to further address this uncertainty.

In conclusion, in this comprehensive Mendelian randomization study of TL and risk for common cancers, we observed a highly significant association between genetic determinants of long TL and increased risk for lung adenocarcinoma. The estimates reported here are not vulnerable to biases caused by reverse causation or confounding by unmeasured environmental factors, strengthening the evidence for a causal role for TL in lung adenocarcinoma. However, the validity of Mendelian randomization estimates is dependent upon several assumptions, namely no pleiotropic effects (independent of TL) of SNPs on the cancer risk or confounders of the TL-cancer relationship. The multi-SNP score for TL should be further investigated as a predictor of adenocarcinoma of the lung, a common lung cancer subtype in both smokers and non-smokers. Future research efforts need to be undertaken to determine the value of telomeres as a novel risk measure or a modifiable pharmacological target, with the long-term goal of improving cancer prediction and prevention.

## Materials and Methods

### The GAME-ON network of consortia for post-GWA research

The goals of the GAME-ON consortium were to pool data from GWA studies to identify new loci, conduct functional studies to identify causal SNPs and biological mechanisms, and to investigate gene–gene and gene–environment interactions as a part of efforts to develop risk prediction models. A secondary goal was to test hypotheses across the centers that might illuminate common mechanisms of susceptibility. Details of GAME-ON and the participating studies are available at http://epi.grants.cancer.gov/gameon/, and described previously (26).

### Identification of SNPs associated with TL

We identified nine SNPs showing independent genome-wide significant associations (*P* < 5 × 10^−8^) with TL in previously published GWA studies among individuals of European ancestry (19–21). Although there are specific cancer susceptibility regions of interest such as the previously described *TERT* locus, our selection of SNPs is based entirely on the SNPs' ability to predict TL based on prior literature, because predictive accuracy is directly related to statistical power for Mendelian randomization (62). The proportion of variance in measured average TL that is explained by individual SNPs ranges from 0.06 to 0.2% (19), and is up to 1.6% for a combined subset of four SNPs (20) (no estimate is currently available for all nine SNPs). Based on the existing literature, we obtained the identifier for the lead SNP at each reported locus as well as the ‘long TL’ allele, association estimate for the ‘long’ allele (in terms of kb increase in TL per allele), standard error and *P*-value for each SNP's association with TL (Table 2). Only the lead SNP from each region was included in the analysis. Although the estimates for these nine SNPs were obtained from three different studies using two different methods (quantitative PCR and Southern blot of the terminal restriction fragment), we scaled the estimates to the same units (kb of TL per risk allele). Comparability between studies is supported by previous studies showing that T/S ratio from qPCR using the Cawthon method is strongly correlated with mean terminal restriction fragment obtained from Southern blot for non-extreme TL values (66). Data on these nine SNPs were available as summary statistics for all cancer types analyzed in the GAME-ON consortium, with the exception of colorectal cancer, for which we obtained proxy SNPs based on strong linkage disequilibrium using the Broad Institute's SNP Annotation and Proxy Search tool (67) (Supplementary Material, Table S4).

### Statistical analysis

For each cancer type, standard fixed-effects meta-analysis methods were used to combine results from individual GWA studies. For each cancer type, genotyping was performed using Illumina or Affymetrix arrays of varying densities described elsewhere (26). Quality control steps taken include gender identity and chromosomal anomaly check, exclusion of related individuals, principal component-based exclusion of individuals of non-European ancestry, exclusion of SNPs and individuals with substantial missingness, exclusion of SNPs in Hardy–Weinberg disequilibrium, and other sample and SNP quality measures. For each study, imputation was performed using the 1000 Genomes Project Phase 1 version 3 reference haplotypes, resulting in up to ∼10 million SNPs being available for the analysis for each cancer type.

Associations between SNPs and cancer risk were estimated using unconditional logistic regression adjusted for age, sex (when applicable) and top principal components (ranging from 2 to 6 across 48 contributing GWA studies). For the lung cancer study, the association was also adjusted for smoking pack-years. We performed the analyses separately for cancer subtypes, including breast (estrogen receptor negative), lung (adenocarcinoma and squamous cell), ovarian (clear cell, endometrioid and serous) and prostate [aggressive and non-aggressive as previously defined (68)]. We performed age- and sex-stratified analyses for overall lung cancer, for which only SNPs imputed to the Illumina 500 K array using the HapMap2 reference panel were available across all sites. For the TL SNPs not available on the 500 K array, we were able to identify tag SNPs (*r*^2^ > 0.8) for all SNPs except PXK SNP rs6772228 (Supplementary Material, Table S5).

We estimated the association between a multi-SNP TL score and risk for each cancer using two different Mendelian randomization methods that require only summarized association estimates for each SNP (hence, no actual score is created for each individual, but we estimated the association that would be observed for such as score if individual-level data were used). This approach is appropriate given that the consortium provides only summary estimates rather than individual-level data for SNP associations. The first method is an inverse-variance weighted average of SNP-specific associations that has been described previously (27,28). The Mendelian randomization estimate ${\hat{\beta }}_{\mathrm{IVW}}$, and its standard error $\text{SE}({\hat{\beta }}_{\mathrm{IVW}})$ were calculated using the following equations:

(1) $${\hat{\beta }}_{\mathrm{IVW}}=\frac{\sum_{k}X_{k}Y_{k}\sigma_{Y_{k}}^{-2}}{\sum_{k}X_{k}^{2}\sigma_{Y_{k}}^{-2}},$$

(2) $$\text{SE(}{\hat{\beta }}_{\mathrm{IVW}})=\sqrt{\frac{1}{\sum_{k}X_{k}^{2}\sigma_{Y_{k}}^{-2}}},$$

where *X_k_* is the per-allele estimate of the *k*th SNP on TL, *Y_k_* is the per-allele estimate of the SNP on the log-odds of cancer and *σ_Yk_* is the corresponding standard error. A schematic summarizing the aforementioned steps is shown in Supplementary Material, Figure S4.

The second method is a likelihood-based method that has been described previously (28). In brief, TL and cancer risk were jointly modeled using a bivariate normal distribution for each genetic variant. The model parameters, including a joint linear effect between TL and cancer log-odds, were estimated using maximum likelihood on the observed data. The likelihood-based analyses were performed using web-based software (http://spark.rstudio.com/sb452/summarized/) (28).

The estimates obtained from the methods described above can be interpreted as the effect of average TL on cancer risk under the following assumptions as previously described for causal inference based on Mendelian randomization (25): (1) the SNPs from the literature are truly predictive of TL in the cancer-prone tissue, (2) the SNPs are not associated with other factors (confounders) that influence both TL and cancer risk and (3) the SNPs only affect cancer risk through their effects on TL, i.e. there are no alternative causal pathways by which the SNPs influence cancer risk.

To visualize the association results for the SNP score, we plotted the association between each SNP and cancer risk against associations with TL (based on the prior literature). Under the assumption that a SNP's association with TL is proportional to its association with cancer risk, one would expect the plotted points to fall along a line that passes through the origin and has a slope equal to the Mendelian randomization estimate. Thus, a steeper slope indicates a stronger magnitude of association between TL and cancer risk. Conversely assuming no causal effect of TL on cancer risk, the Mendelian randomization estimate would correspond to a slope of zero.

To assess a potential violation of the first assumption (i.e., a true association between each of the variant and TL), an additional analysis was performed after excluding the SNP near the *PXK* region (rs6772228), which may be a false-positive association evidenced by its lack of plausible biological explanation, and the lack of consistency in its association with TL across several study sites (21). This analysis is referred to as the ‘strict’ analysis. To assess potential violation of the second and third assumptions (i.e., no confounding or pleiotropy), a goodness-of-fit test was performed for each SNP set under the null hypothesis that each SNP included in the SNP score has an association with cancer risk that is proportional to its association with TL. The rejection of the null hypothesis indicated heterogeneity of the associations between SNPs and cancer risk relative to the associations between the SNPs and TL. In instances where the null hypothesis was rejected, stepwise removal of SNPs from the SNP set was performed until there was no significant heterogeneity, based on a method previously described (http://cran.r-project.org/web/packages/gtx/) (29). Specifically, For *K* uncorrelated SNPs,

(3) $$X_{k}^{2}=\underset{k}{\sum }Y_{k}^{2}\sigma_{Y_{k}}^{-2},$$

(4) $$X_{\mathrm{rs}}^{2}={\left(\frac{{\hat{\beta }}_{\mathrm{IVW}}}{\text{SE(}{\hat{\beta }}_{\mathrm{IVW}})}\right)}^{2},$$

in which $X_{k}^{2}$ and $X_{\mathrm{rs}}^{2}$ are $\chi_{\left(k\right)}^{2}$ and $\chi_{\left(1\right)}^{2}$ distributed test statistics, respectively, for the association between each cancer type and all *K* SNPs under an unconstrained *K* degree-of-freedom model, and for the nested 1 degree-of-freedom risk score model, respectively. Then the goodness-of-fit test statistic is

(5) $$Q_{\mathrm{rs}}=X_{k}^{2}-X_{\mathrm{rs}}^{2},$$

in which *Q*_rs_ is $\chi_{\left(k-1\right)}^{2}$ distributed under the null hypothesis that all *K* SNPs are associated with cancer risk with true association sizes proportional to the associations with TL. For each analysis in which the goodness-of-fit test null hypothesis was rejected (*P* < 0.05), we removed the SNP that resulted in the greatest reduction of the *Q*_rs_ test statistic, and repeated the goodness-of-fit test. If still *P* < 0.05, we repeated the exclusion procedure until *P* > 0.05. The association estimates obtained from the subsequent analysis using the remaining SNPs that pass the goodness-of-fit test is referred to as the ‘goodness-of-fit based’ estimates and displayed in Supplementary Material, Table S1. *P*-values for the goodness-of-fit test before and after exclusion of SNPs, and which SNPs were excluded for each cancer analysis for the ‘goodness-of-fit based’ analysis are shown in Supplementary Material, Table S2. An example for this procedure is as follows: for lung adenocarcinoma, the inclusion of all nine SNPs results in an OR of 2.87 (*P* = 6.3 × 10^−15^), but the goodness-of-fit test *P*-value of 9.0 × 10^−6^ indicates the presence of heterogeneity with at least one of the SNPs used in the SNP score. The *TERT* SNP was identified to be the SNP contributing the most to this heterogeneity (by stepwise exclusion), and once removed, resulted in a SNP score with a goodness-of-fit *P*-value of 0.09, indicating a lack of substantial heterogeneity in ratio of SNP–cancer to SNP–telomere associations across SNPs. After exclusion of the *TERT* SNP, we still observe a significant association in the same direction, albeit attenuated (OR = 2.00, *P* = 6.6 × 10^−6^).

We performed power analyses to estimate the minimum detectable magnitude of association for each cancer type given the sample sizes available in the GAME-ON study, in terms of OR per 1 kb increase in TL. This was done using a web-based application (http://glimmer.rstudio.com/kn3in/mRnd/) (69), specifying 80% power, 0.05 type I error rate, and assuming the variance in TL explained by the nine SNPs is *R*^2^ = 0.01 or *R*^2^ = 0.02, respectively. Because the web application calculates the detectable OR of cancer risk per one standard deviation of TL, which is roughly equivalent to 500 bp (36,37), we exponentiated this OR to the power of 0.5 in order to obtain the detectable OR per 1000 bp increase TL. (Supplementary Material, Table S3).

## Supplementary Material

Supplementary Material is available at *HMG* online.

## Funding

This work was supported by the Genetic Associations and Mechanisms in Oncology Network, GAME-ON: Discovery, Biology, and Risk of Inherited Variants in Breast Cancer, DRIVE, PI: D.J.H. (U19 CA148065); Colorectal Transdisciplinary Study, CORECT, PI: S.B.G. (U19 CA148107); Transdisciplinary Research in Cancer of the Lung, TRICL, PI: C.A. (U19 CA148127); Follow-up of ovarian cancer genetic association and interaction studies, FOCI, PI: T.A.S. (U19 CA148112); Elucidating Loci Involved in Prostate Cancer Susceptibility, ELLIPSE, PI: B.E.H. (U19 CA148537); Genetics and Epidemiology of Colorectal Cancer Consortium, GECCO: National Cancer Institute, National Institutes of Health, US Department of Health and Human Services (U01 CA137088, R01 CA059045); National Institute of Health, National Institute on Aging (T32AG000243; P30AG012857) to C.Z.; Cancer Research Foundation Young Investigator Award, R01 ES020506, and U01 HG007601 to BLP; The Wellcome Trust (100114) to S.B.; The NHMRC Senior Principal Research Fellow to G.C.T. Funding to pay the Open Access publication charges for this article was provided by the University of Cambridge (COAF Open Access block grant).

*Conflict of Interest statement*. None declared

## Supplementary Material

Supplementary Data
