# Supplementary material for: Genetic determinants of telomere length and risk of common cancers: a Mendelian randomization study
Source: Hum Mol Genet. 2015 Jul 2;24(18):5356–66. doi: 10.1093/hmg/ddv252 (PMC4550826; doi:10.1093/hmg/ddv252)
Supplement: Supplementary Data [file supp_24_18_5356__index.html]

Genetic determinants of telomere length and risk of common cancers: a Mendelian randomization study — Genetic determinants of telomere length and risk of common cancers: a Mendelian randomization study — Genetic determinants of telomere length and risk of common cancers: a Mendelian randomization study — Supplementary Data 

# Genetic determinants of telomere length and risk of common cancers: a Mendelian randomization study

## Supplementary Data

Supplementary Data

- Supplementary Data - Docx file
