# Supplementary material for: Genetic determinants of telomere length and risk of common cancers: a Mendelian randomization study
Source: Hum Mol Genet. 2015 Jul 2;24(18):5356–66. doi: 10.1093/hmg/ddv252 (PMC4550826; doi:10.1093/hmg/ddv252)
Supplement: Supplementary Data [file supp_ddv252_ddv252supp.docx]

Supplementary Figure Legends

**Supplementary Figure 1.** Forest plots of association estimates (with horizontal bars indicating 95% confidence interval) for the “long telomere” allele of each SNP with cancer risk. SNPs are ordered by increasing magnitude of association with telomere length

**Supplementary Figure 2.** Scatter plots showing the per-allele association with cancer risk plotted against the per-allele association with kb of telomere length (with vertical and horizontal black lines showing 95% confidence interval for each SNP). The scatter plot is overlaid with the Mendelian randomization estimate (slope of red solid line with dotted lines showing 95% confidence interval) of the effect of TL on cancer risk

**Supplementary Figure 3.** Forest plot (top) and scatter plot (bottom) of associations between telomere length-associated SNPs and risk for colorectal cancer. Forest plots show association estimates (with horizontal bars indicating 95% confidence interval) for the “long telomere” allele of each SNP with cancer risk. SNPs are ordered by increasing magnitude of association with telomere length. Scatter plots show the per-allele association with cancer risk plotted against the per-allele association with kb of telomere length (with vertical and horizontal black lines showing 95% confidence interval for each SNP). The scatter plot is overlaid with the Mendelian randomization estimate (slope of red solid line with dotted lines showing 95% confidence interval) of the effect of TL on cancer risk Data is from 9,488 independent cases and 12,040 controls from the Genetics and Epidemiology of Colorectal Cancer Consortium (GECCO)

**Supplementary Figure 4.** Schematic of the Mendelian randomization estimation method using data from two different sources

**Supplementary Figure 1.** Forest plots of association estimates (with horizontal bars indicating 95% confidence interval) for the “long telomere” allele of each SNP with cancer risk. SNPs are ordered by increasing magnitude of association with telomere length


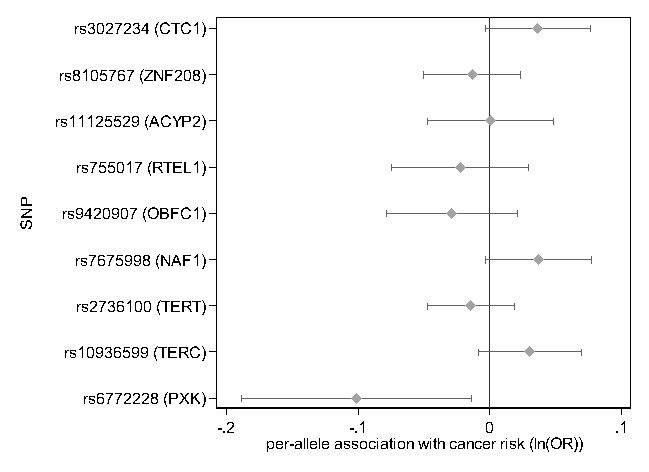

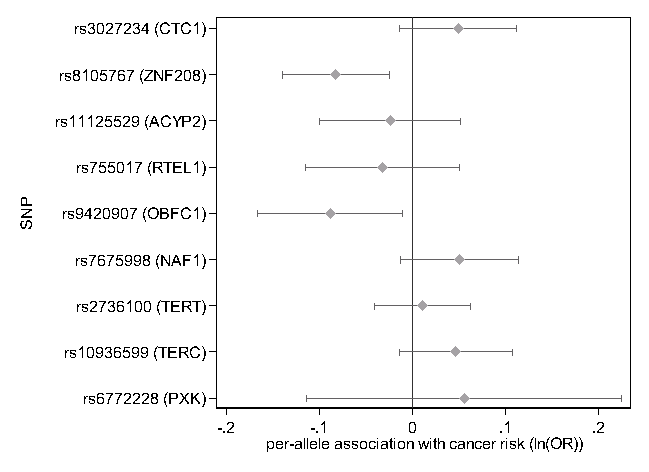

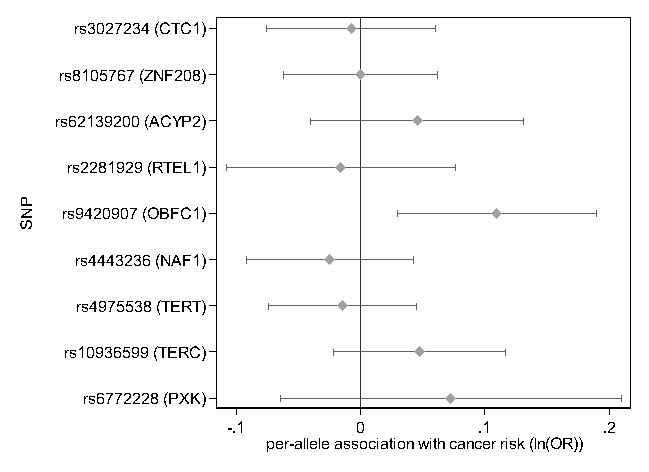

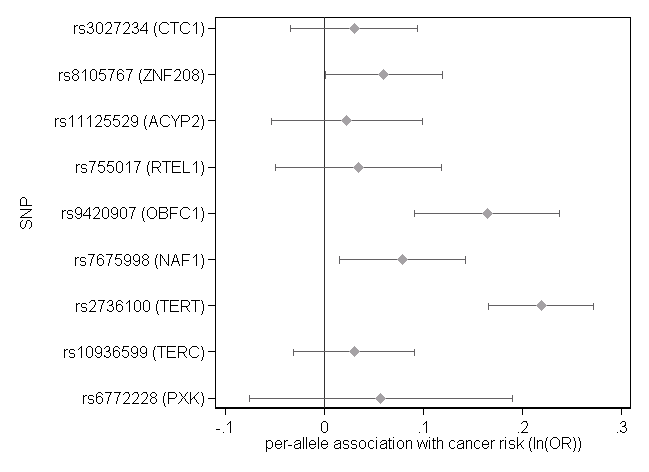

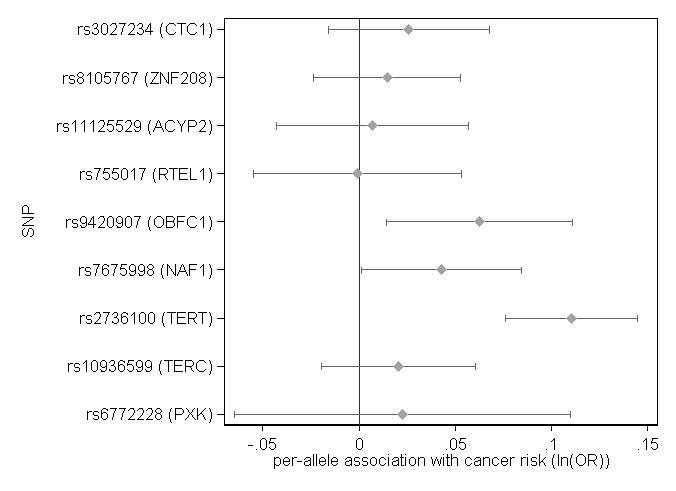

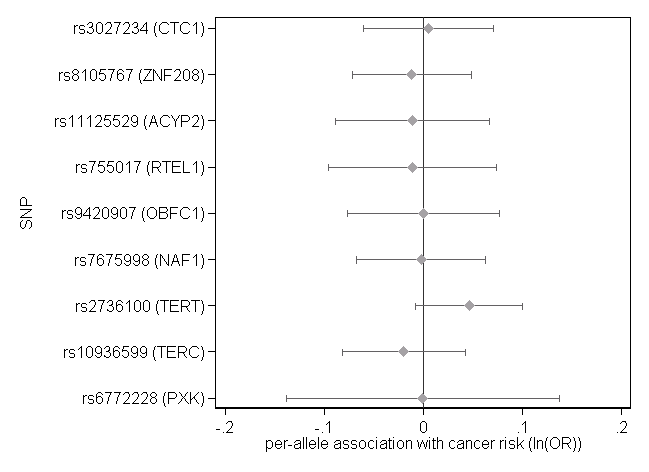


Breast cancer – ER-negative

Breast cancer – all subtypes

Colorectal cancer – all subtypes

Lung cancer – all subtypes

Lung cancer - adenocarcinoma

Lung cancer - squamous

**Supplementary Figure 1.** Continued


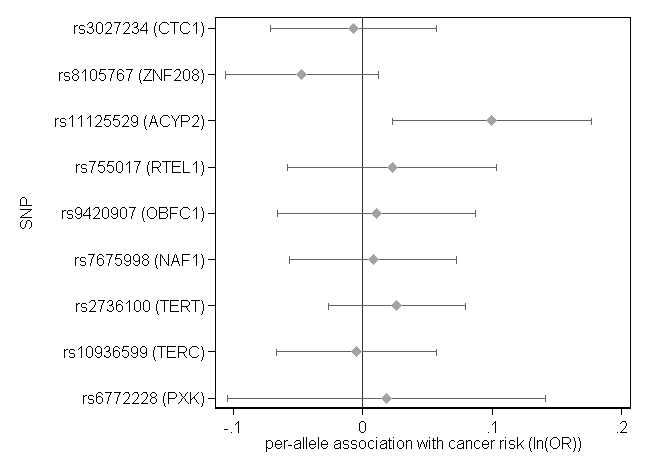

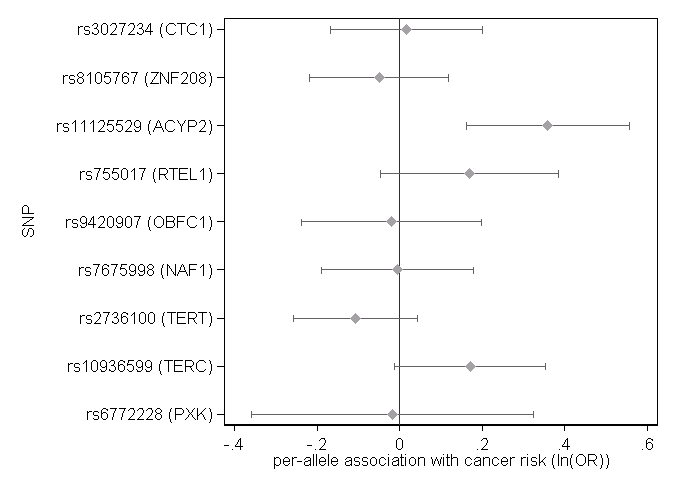

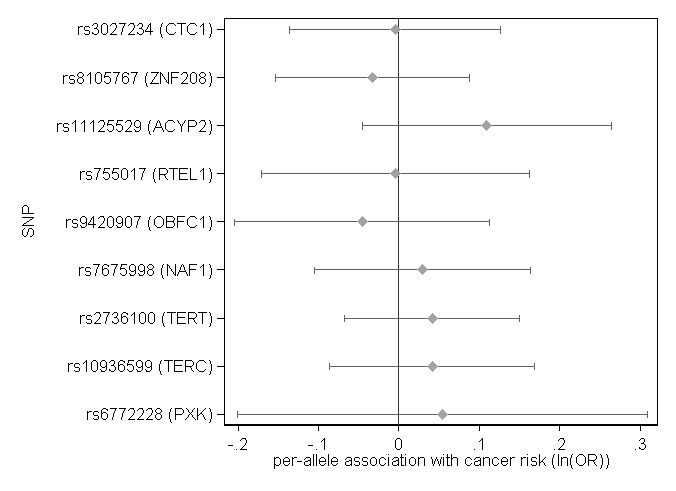

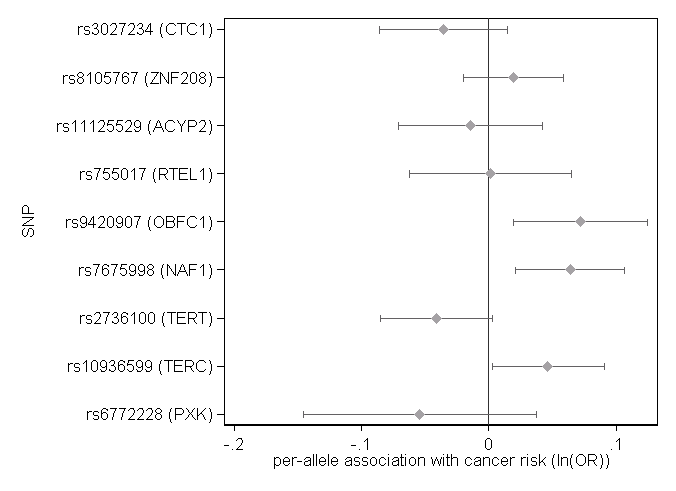

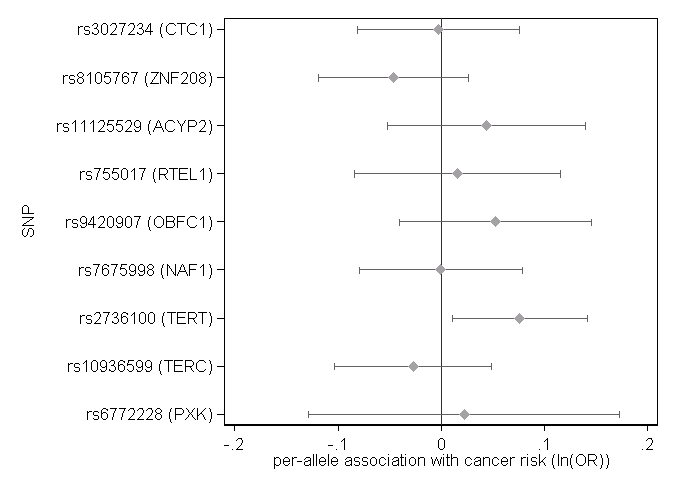

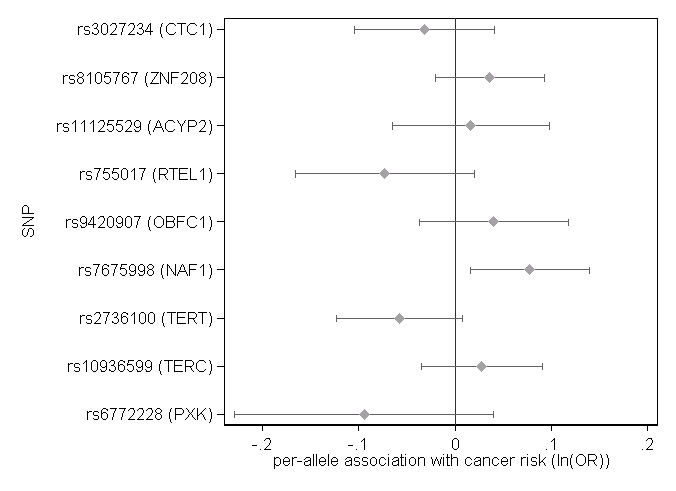


Ovarian cancer – clear cell

Ovarian cancer – all subtypes

Ovarian cancer – endometrioid

Ovarian cancer – serous

Prostate cancer – all subtypes

Prostate cancer - aggressive

**Supplementary Figure 2.** Scatter plots showing the per-allele association with cancer risk plotted against the per-allele association with kb of telomere length (with vertical and horizontal black lines showing 95% confidence interval for each SNP). The scatter plot is overlaid with the Mendelian randomization estimate (slope of red solid line with dotted lines showing 95% confidence interval) of the effect of TL on cancer risk


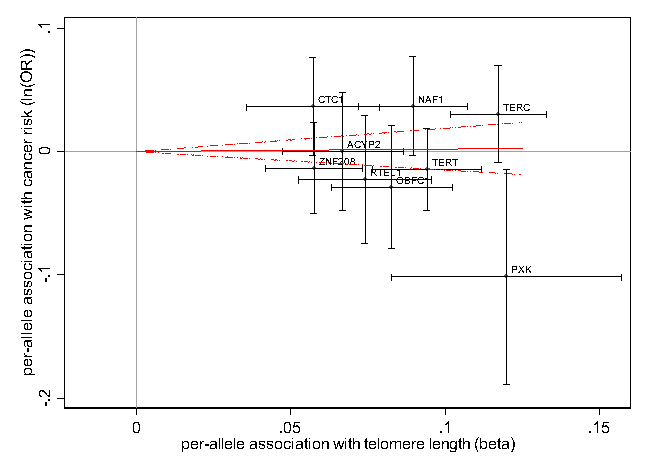

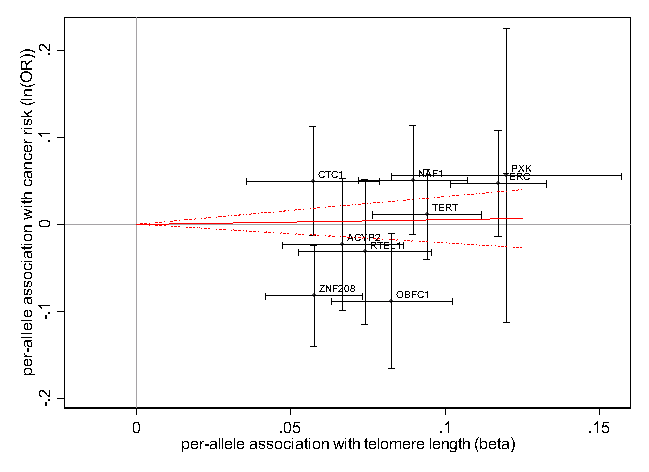

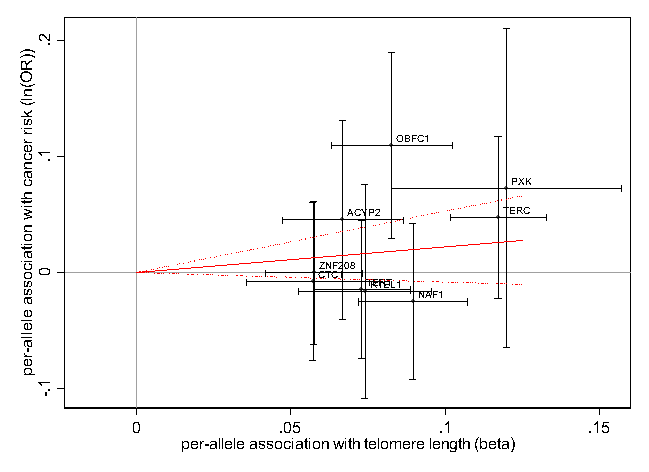

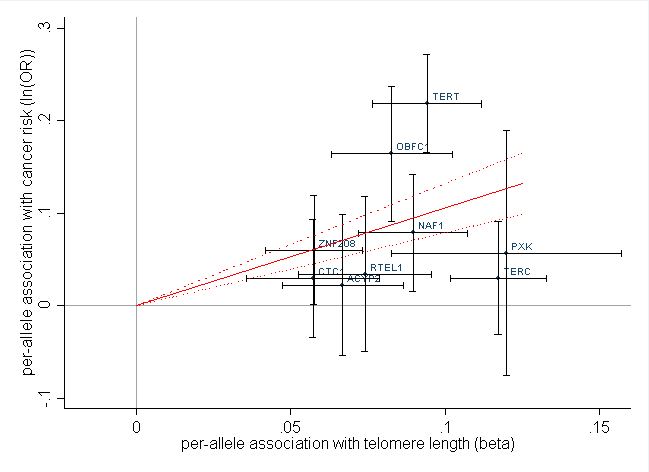

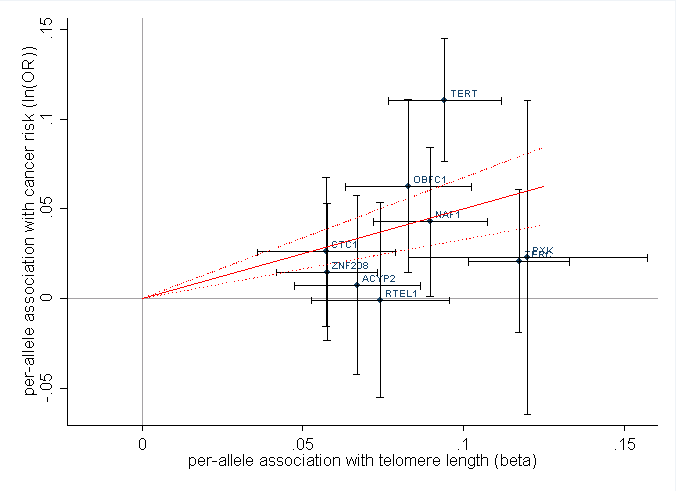

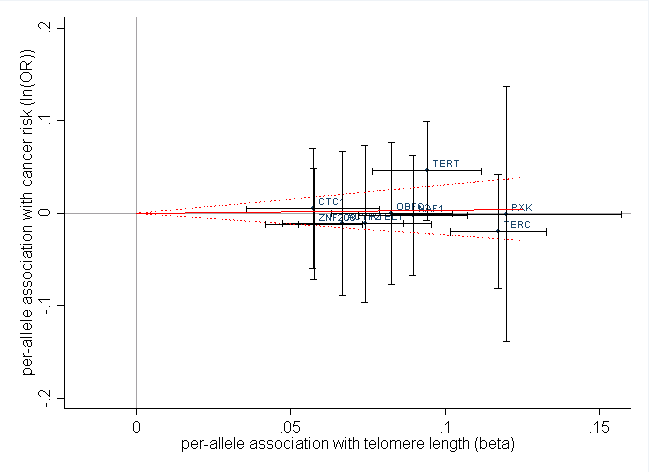


Breast cancer – ER-negative

Breast cancer – all subtypes

Colorectal cancer – all subtypes

Lung cancer – all subtypes

Lung cancer - adenocarcinoma

Lung cancer - squamous

**Supplementary Figure 2.** Continued


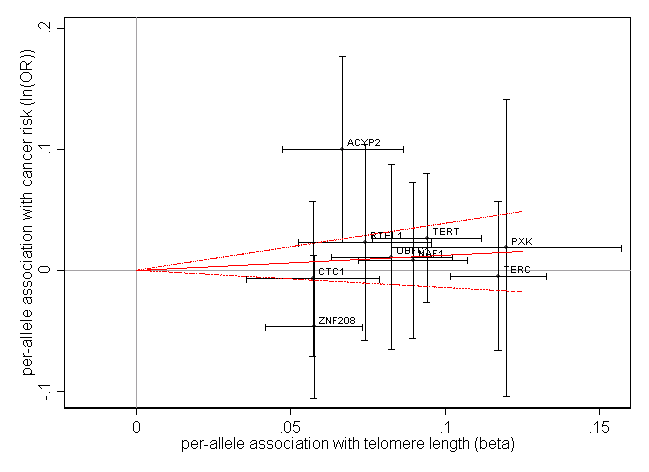

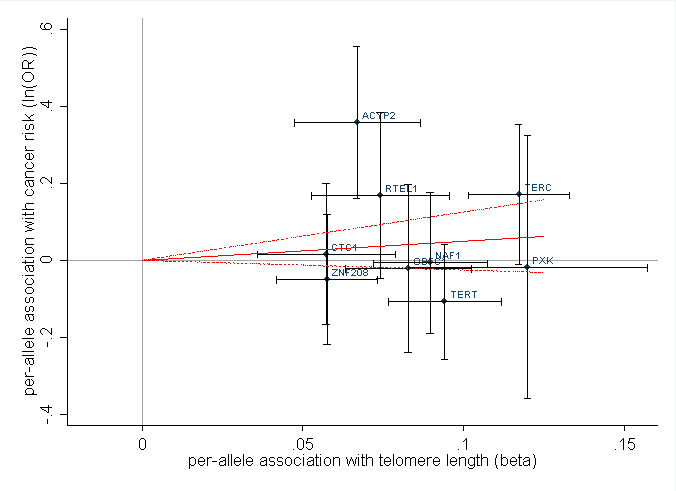

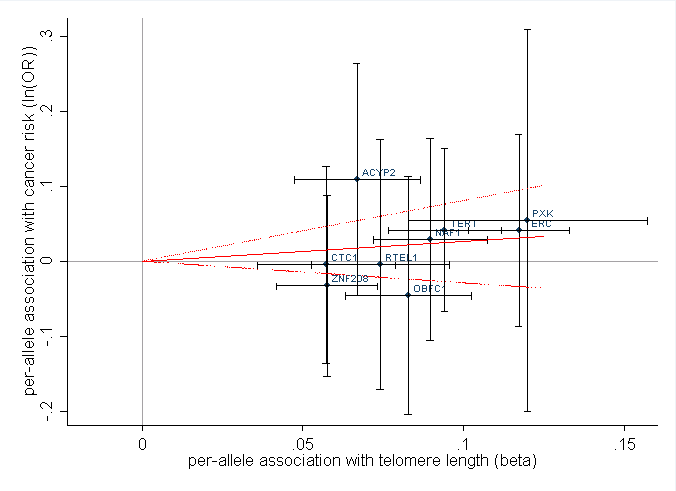

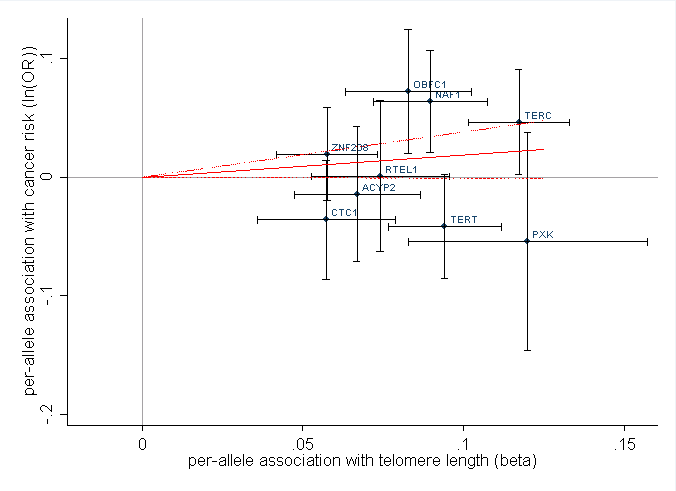

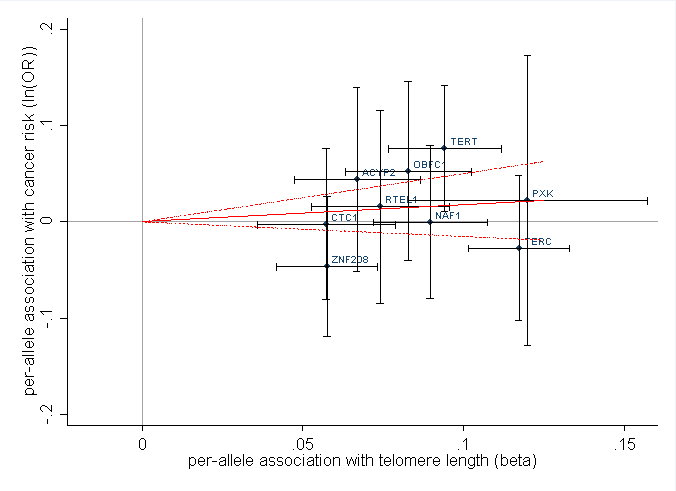

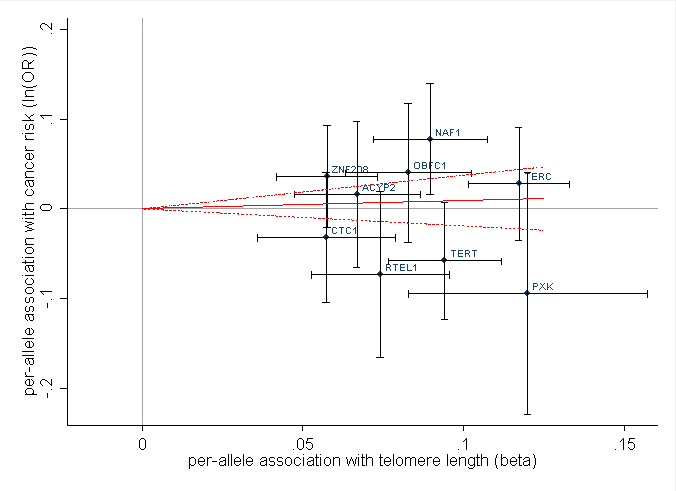


Ovarian cancer – clear cell

Ovarian cancer – all subtypes

Ovarian cancer – endometrioid

Ovarian cancer – serous

Prostate cancer – all subtypes

Prostate cancer - aggressive

**Supplementary Figure 3.** Forest plot (top) and scatter plot (bottom) of associations between telomere length-associated SNPs and risk for colorectal cancer. Forest plots show association estimates (with horizontal bars indicating 95% confidence interval) for the “long telomere” allele of each SNP with cancer risk. SNPs are ordered by increasing magnitude of association with telomere length. Scatter plots show the per-allele association with cancer risk plotted against the per-allele association with kb of telomere length (with vertical and horizontal black lines showing 95% confidence interval for each SNP). The scatter plot is overlaid with the Mendelian randomization estimate (slope of red solid line with dotted lines showing 95% confidence interval) of the effect of TL on cancer risk Data is from 10,314 independent cases and 12,857 controls from the Genetics and Epidemiology of Colorectal Cancer Consortium (GECCO)


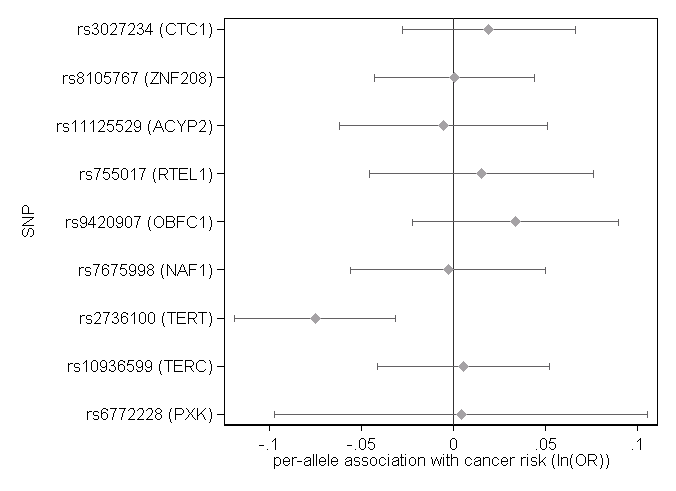

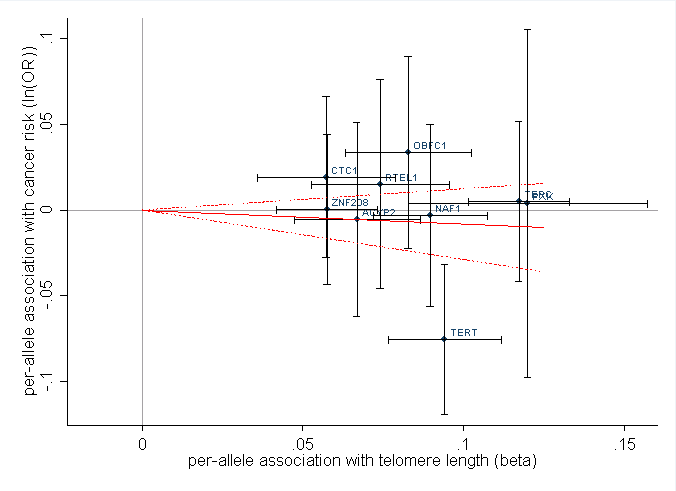


**Supplementary Figure 4.** Schematic of the Mendelian randomization estimation method using data from two different sources

Apply equations (1) and (2) (Methods)

From literature:

Obtain X_k_, the per-allele association for the k-th SNP

**SNP**

**Telomere Length**

**X_k_**

From GAME-ON:

Obtain Y_k_, the per-allele association for the k-th SNP

**SNP**

**Cancer Risk**

**Y_k_**

Estimated:

Estimate $\hat{\beta}_{IVW}$, the multi-SNP risk score association, corresponding to the effect of telomere length on cancer risk under Mendelian randomization assumptions

**Telomere Length**

**Cancer Risk**

$$\hat{\beta}_{IVW}$$

**Supplementary Table 1.** Odds ratios of cancer risk per 1000 base pair increase in telomere length according to a multi-SNP telomere length score analysis of strict^a^, goodness-of-fit (GoF) based^b^, and strict-goodness-of-fit based^c^ list of SNPs.

|  | **Strict** | | |  | | |  | **GoF-based** | | | |  | **Strict + GoF-based** | | | | | |  |
| --- | --- | --- | --- | --- | --- | --- | --- | --- | --- | --- | --- | --- | --- | --- | --- | --- | --- | --- | --- |
| **Cancer Type** | **Estimate** | **95% CI** | **P_fit_** | |  | | **Estimate** | | | **95% CI** | **P_fit_** | | |  | **Estimate** | **95% CI** | **P_fit_** |  |  |
| **Breast** |  |  |  | | |  | | |  |  |  | | |  |  |  |  |  |  |
| All | 1.07 | 0.90, 1.28 | 0.43 | | |  | | | 1.07 | 0.90, 1.28 | 0.43 | | |  | 1.07 | 0.90, 1.28 | 0.43 |  |  |
| ER-negative | 1.04 | 0.79, 1.36 | 0.79 | | |  | | | 1.18 | 0.89, 1.55 | 0.24 | | |  | 1.16 | 0.88, 1.54 | 0.29 |  |  |
| **Colorectal** | 1.21 | 0.89, 1.66 | 0.23 | | |  | | | 1.25 | 0.92, 1.69 | 0.15 | | |  | 1.21 | 0.89, 1.66 | 0.23 |  |  |
| **Lung** |  |  |  | | |  | | |  |  |  | | |  |  |  |  |  |  |
| All | 1.68 | 1.41, 2.01 | 1.0x10^-8^ | | |  | | | 1.36 | 1.12, 1.65 | 2.3x10^-3^ | | |  | 1.37 | 1.12, 1.68 | 2.5x10^-3^ |  |  |
| Adeno-carcinoma | 2.93 | 2.26, 3.80 | 5.9x10^-16^ | | |  | | | 2.00 | 1.48, 2.70 | 6.6x10^-6^ | | |  | 2.06 | 1.53, 2.78 | 1.9x10^-6^ |  |  |
| Squamous | 0.98 | 0.74, 1.29 | 0.87 | | |  | | | 1.04 | 0.79, 1.36 | 0.79 | | |  | 0.98 | 0.74, 1.29 | 0.87 |  |  |
| **Ovarian** |  |  |  | | |  | | |  |  |  | | |  |  |  |  |  |  |
| All | 1.13 | 0.86, 1.48 | 0.39 | | |  | | | 1.13 | 0.87, 1.47 | 0.37 | | |  | 1.13 | 0.86, 1.48 | 0.39 |  |  |
| Clear-cell | 1.73 | 0.79, 3.79 | 0.17 | | |  | | | 1.18 | 0.54, 2.57 | 0.68 | | |  | 1.21 | 0.54, 2.72 | 0.65 |  |  |
| Endometrioid | 1.28 | 0.73, 2.25 | 0.40 | | |  | | | 1.30 | 0.75, 2.24 | 0.35 | | |  | 1.28 | 0.73, 2.25 | 0.40 |  |  |
| Serous | 1.19 | 0.85, 1.67 | 0.32 | | |  | | | 1.19 | 0.86, 1.65 | 0.30 | | |  | 1.19 | 0.85, 1.67 | 0.32 |  |  |
| **Prostate** |  |  |  | | |  | | |  |  |  | | |  |  |  |  |  |  |
| All | 1.26 | 1.03, 1.54 | 0.02 | | |  | | | 1.46 | 1.17, 1.82 | 7.9x10^-4^ | | |  | 1.47 | 1.17, 1.83 | 7.5x10^-4^ |  |  |
| Aggressive | 1.16 | 0.87, 1.56 | 0.31 | | |  | | | 0.94 | 0.69, 1.28 | 0.68 | | |  | 1.37 | 0.99, 1.89 | 0.05 |  |  |

^a^Strict set includes only high-confidence SNPs.

^b^GoF-based set includes only non-pleiotropic SNPs based on goodness-of-fit test

^c^Strict-GoF-based set includes only high-confidence, non-pleiotropic SNPs

**Supplementary Table 2.** P-values (P_fit_) for the goodness-of-fit test before and after exclusion of SNPs, and the SNPs excluded for each cancer analysis for the “goodness-of-fit based^a^” analysis.

| **Cancer Type** | **P-value of goodness-of-fit test statistic including all SNPs** | **SNPs excluded in stepwise procedure** | **P-value of the goodness-of-fit test statistic after SNP exclusion** |
| --- | --- | --- | --- |
| **Breast** |  |  |  |
| All | 0.03 | rs3027234 (PXK) | 0.12 |
| ER-negative | 6.5x10^-3^ | rs8105767 (ZNF208) | 0.09 |
| **Colorectal** | 0.22 | - | - |
| **Lung** |  |  |  |
| All | 5.8x10^-3^ | rs2736100 (TERT) | 0.70 |
| Adenocarcinoma | 9.0x10^-6^ | rs2736100 (TERT) | 0.09 |
| Squamous | 0.90 | - | - |
| **Ovarian** |  |  |  |
| All | 0.29 | - | - |
| Clear-cell | 0.01 | rs11125529 (ACYP2) | 0.34 |
| Endometrioid | 0.94 | - | - |
| Serous | 0.40 | - | - |
| **Prostate** |  |  |  |
| All | 2.0x10^-3^ | rs2736100, rs3027234  (TERT, CTC1) | 0.08 |
| Aggressive | 0.03 | rs7675998 (NAF1) | 0.13 |

^a^The goodness-of-fit test described by Johnson (http://cran.r-project.org/web/packages/gtx/index.html) was applied to the nine SNPs used in the multi-SNP score for each cancer risk association analysis. A chi-squared test statistic was calculated under the null hypothesis that all nine SNPs are associated with cancer risk with true effect sizes proportional to the effects on telomere length. For each analysis in which the goodness-of-fit test null hypothesis was rejected (p<0.05), we removed the SNP that resulted in the greatest reduction of the test statistic. For all but one cancer subtype analysis, the exclusion of one SNP removed evidence of heterogeneity (p>0.05). For the overall prostate cancer analysis, a second SNP was excluded based on the above described method, resulting in no remaining evidence of heterogeneity. All remaining SNPs that passed the “goodness-of-fit” test was evaluated as multi-SNP scores in association with cancer risk.

**Supplementary Table 3.** Approximate odds ratio (OR) detectable per 2 standard deviations of telomere length (1000 bp) given sample size, case proportion, and 80% power with 0.05 type-I error rate assuming the variance in TL explained by the nine SNPs is R^2^=0.01 or R^2^=0.02, respectively.

| **Cancer Type** | **Sample size** | **Proportion cases** | **OR (R^2^=0.01)** | **OR (R^2^=0.02)** |
| --- | --- | --- | --- | --- |
| **Breast** |  |  |  |  |
| All | 33,832 | 0.47 | 0.86/1.16 | 0.90/1.11 |
| ER-negative | 18,067 | 0.27 | 0.81/1.24 | 0.85/1.17 |
|  |  |  |  |  |
| **Colorectal** | 9,931 | 0.51 | 0.76/1.32 | 0.82/1.22 |
| **Colorectal (GECCO)** | 21,528 | 0.44 | 0.83/1.20 | 0.88/1.14 |
|  |  |  |  |  |
| **Lung^a^** |  |  |  |  |
| All | 28,998 | 0.42 | 0.85/1.17 | 0.89/1.12 |
| Adenocarcinoma | 19,589 | 0.19 | 0.80/1.24 | 0.85/1.17 |
| Squamous | 19,437 | 0.18 | 0.80/1.25 | 0.85/1.18 |
|  |  |  |  |  |
| **Ovarian^a^** |  |  |  |  |
| All | 13,492 | 0.55 | 0.79/1.4 | 0.84/1.17 |
| Clear-cell | 9,479 | 0.10 | 0.63/1.62 | 0.70/1.44 |
| Endometrioid | 9,836 | 0.18 | 0.68/1.52 | 0.74/1.35 |
| Serous | 11,696 | 0.44 | 0.77/1.41 | 0.82/1.22 |
|  |  |  |  |  |
| **Prostate** |  |  |  |  |
| All | 26,884 | 0.53 | 0.84/1.19 | 0.88/1.13 |
| Aggressive | 17,174 | 0.26 | 0.80/1.24 | 0.85/1.17 |

**Supplementary Table 4.** Proxy SNPs of genetic variants associated with long relative telomere length from literature used for colorectal cancer association analyses

| Chromosome | Locus | Original SNP | Proxy SNP | Distance | r^2^ | Proxy Long Allele |
| --- | --- | --- | --- | --- | --- | --- |
| 2 | ACYP2 | rs11125529 | rs62139200 | 359 | 1.000 | T |
| 4 | NAF1 | rs7675998 | rs4443236 | 3316 | 0.953 | T |
| 5 | TERT | rs7726159 | rs4975538 | 1489 | 0.930 | C |
| 20 | RTEL1 | rs755017 | rs2281929 | 458 | 1.000 | C |

**Supplementary Table 5.** Proxy SNPs of genetic variants associated with long relative telomere length from literature used for age and sex stratified overall lung cancer association analyses

| Chromosome | Locus | Original SNP | Proxy SNP | Distance | r^2^ | Proxy Long Allele |
| --- | --- | --- | --- | --- | --- | --- |
| 4 | NAF1 | rs7675998 | rs11100479 | 5081 | 0.953 | T |
| 19 | ZNF208 | rs8105767 | rs7257051 | 103851 | 0.853 | G |
| 20 | RTEL1 | rs755017 | rs2281929 | 458 | 1.000 | C |
| 2 | ACYP2 | rs11125529 | rs11890390 | 9816 | 0.932 | T |
| 17 | CTC1 | rs3027234 | rs8075078 | 50004 | 0.862 | G |

**Funding and Acknowledgements:**

**BCFR:**

The Breast Cancer Family Registry (BCFR) is supported by grant UM1 CA164920 from the USA National Cancer Institute. The content of this manuscript does not necessarily reflect the views or policies of the National Cancer Institute or any of the collaborating centers in the Breast Cancer Family Registry (BCFR), nor does mention of trade names, commercial products, or organizations imply endorsement by the USA Government or the BCFR.

**GECCO:**

The authors would like to thank all those at the GECCO Coordinating Center for helping bring together the data and people that made this project possible. The authors acknowledge Dave Duggan and team members at TGEN (Translational Genomics Research Institute), the Broad Institute, and the Génome Québec Innovation Center for genotyping DNA samples of cases and controls, and for scientific input for GECCO.

ASTERISK: a Hospital Clinical Research Program (PHRC) and supported by the Regional Council of Pays de la Loire, the Groupement des Entreprises Françaises dans la Lutte contre le Cancer (GEFLUC), the Association Anne de Bretagne Génétique and the Ligue Régionale Contre le Cancer (LRCC). We are very grateful to Dr. Bruno Buecher without whom this project would not have existed. We also thank all those who agreed to participate in this study, including the patients and the healthy control persons, as well as all the physicians, technicians and students.

COLO2&3: National Institutes of Health (R01 CA60987).

DACHS: German Research Council (Deutsche Forschungsgemeinschaft, BR 1704/6-1, BR 1704/6-3, BR 1704/6-4 and CH 117/1-1), and the German Federal Ministry of Education and Research (01KH0404 and 01ER0814). We thank all participants and cooperating clinicians, and Ute Handte-Daub, Renate Hettler-Jensen, Utz Benscheid, Muhabbet Celik and Ursula Eilber for excellent technical assistance.

DALS: National Institutes of Health (R01 CA48998 to M.L.S);

HPFS is supported by the National Institutes of Health (P01 CA 055075, UM1 CA167552, R01 137178, R01 CA 151993 and P50 CA 127003), NHS by the National Institutes of Health (R01 CA137178, P01 CA 087969, R01 CA151993 and P50 CA 127003,) and PHS by the National Institutes of Health (R01 CA042182). We would like to acknowledge Patrice Soule and Hardeep Ranu of the Dana Farber Harvard Cancer Center High-Throughput Polymorphism Core who assisted in the genotyping for NHS, HPFS, and PHS under the supervision of Dr. Immaculata Devivo and Dr. David Hunter, Qin (Carolyn) Guo and Lixue Zhu who assisted in programming for NHS and HPFS, and Haiyan Zhang who assisted in programming for the PHS. We would like to thank the participants and staff of the Nurses' Health Study and the Health Professionals Follow-Up Study, for their valuable contributions as well as the following state cancer registries for their help: AL, AZ, AR, CA, CO, CT, DE, FL, GA, ID, IL, IN, IA, KY, LA, ME, MD, MA, MI, NE, NH, NJ, NY, NC, ND, OH, OK, OR, PA, RI, SC, TN, TX, VA, WA, WY. The authors assume full responsibility for analyses and interpretation of these data.

MEC: National Institutes of Health (R37 CA54281, P01 CA033619, and R01 CA63464).

OFCCR: National Institutes of Health, through funding allocated to the Ontario Registry for Studies of Familial Colorectal Cancer (U01 CA074783); see CCFR section above. Additional funding toward genetic analyses of OFCCR includes the Ontario Research Fund, the Canadian Institutes of Health Research, and the Ontario Institute for Cancer Research, through generous support from the Ontario Ministry of Research and Innovation.

PLCO: Intramural Research Program of the Division of Cancer Epidemiology and Genetics and supported by contracts from the Division of Cancer Prevention, National Cancer Institute, NIH, DHHS. Additionally, a subset of control samples were genotyped as part of the Cancer Genetic Markers of Susceptibility (CGEMS) Prostate Cancer GWAS (Yeager, M et al. Genome-wide association study of prostate cancer identifies a second risk locus at 8q24. Nat Genet 2007 May;39(5):645-9), Colon CGEMS pancreatic cancer scan (PanScan) (Amundadottir, L et al. Genome-wide association study identifies variants in the ABO locus associated with susceptibility to pancreatic cancer. Nat Genet. 2009 Sep;41(9):986-90, and Petersen, GM et al. A genome-wide association study identifies pancreatic cancer susceptibility loci on chromosomes 13q22.1, 1q32.1 and 5p15.33. Nat Genet. 2010 Mar;42(3):224-8), and the Lung Cancer and Smoking study (Landi MT, et al. A genome-wide association study of lung cancer identifies a region of chromosome 5p15 associated with risk for adenocarcinoma. Am J Hum Genet. 2009 Nov;85(5):679-91). The prostate and PanScan study datasets were accessed with appropriate approval through the dbGaP online resource (http://cgems.cancer.gov/data/) accession numbers phs000207.v1.p1 and phs000206.v3.p2, respectively, and the lung datasets were accessed from the dbGaP website (http://www.ncbi.nlm.nih.gov/gap) through accession number phs000093.v2.p2. Funding for the Lung Cancer and Smoking study was provided by National Institutes of Health (NIH), Genes, Environment and Health Initiative (GEI) Z01 CP 010200, NIH U01 HG004446, and NIH GEI U01 HG 004438. For the lung study, the GENEVA Coordinating Center provided assistance with genotype cleaning and general study coordination, and the Johns Hopkins University Center for Inherited Disease Research conducted genotyping. The authors thank Drs. Christine Berg and Philip Prorok, Division of Cancer Prevention, National Cancer Institute, the Screening Center investigators and staff or the Prostate, Lung, Colorectal, and Ovarian (PLCO) Cancer Screening Trial, Mr. Tom Riley and staff, Information Management Services, Inc., Ms. Barbara O’Brien and staff, Westat, Inc., and Drs. Bill Kopp, Wen Shao, and staff, SAIC-Frederick. Most importantly, we acknowledge the study participants for their contributions to making this study possible. The statements contained herein are solely those of the authors and do not represent or imply concurrence or endorsement by NCI.

PMH: National Institutes of Health (R01 CA076366 to P.A. Newcomb). The authors would like to thank the study participants and staff of the Hormones and Colon Cancer study.

VITAL: National Institutes of Health (K05 CA154337).

WHI: The WHI program is funded by the National Heart, Lung, and Blood Institute, National Institutes of Health, U.S. Department of Health and Human Services through contracts HHSN268201100046C, HHSN268201100001C, HHSN268201100002C, HHSN268201100003C, HHSN268201100004C, and HHSN271201100004C. The authors thank the WHI investigators and staff for their dedication, and the study participants for making the program possible. A full listing of WHI investigators can be found at: http://www.whi.org/researchers/Documents%20%20Write%20a%20Paper/WHI%20Investigator%20Short%20List.pdf
